# Dietary Treatment of Metabolic Acidosis in Chronic Kidney Disease

**DOI:** 10.3390/nu10040512

**Published:** 2018-04-20

**Authors:** Roswitha Siener

**Affiliations:** University Stone Centre, Department of Urology, University of Bonn, Sigmund-Freud-Straße 25, D-53105 Bonn, Germany; Roswitha.Siener@ukbonn.de; Tel.: +49-228-2871-9034

**Keywords:** metabolic acidosis, kidney disease, urolithiasis, urinary stones, protein, fruits, vegetables, bicarbonate, alkali citrate

## Abstract

Chronic kidney disease and reduced glomerular filtration rate are risk factors for the development of chronic metabolic acidosis. The prevention or correction of chronic metabolic acidosis has been found to slow progression of chronic kidney disease. Dietary composition can strongly affect acid–base balance. Major determinants of net endogenous acid production are the generation of large amounts of hydrogen ions, mostly by animal-derived protein, which is counterbalanced by the metabolism of base-producing foods like fruits and vegetables. Alkali therapy of chronic metabolic acidosis can be achieved by providing an alkali-rich diet or oral administration of alkali salts. The primary goal of dietary treatment should be to increase the proportion of fruits and vegetables and to reduce the daily protein intake to 0.8–1.0 g per kg body weight. Diet modifications should begin early, i.e., even in patients with moderate kidney impairment, because usual dietary habits of many developed societies contribute an increased proportion of acid equivalents due to the high intake of protein from animal sources.

## 1. Introduction

The kidney plays an important part in the regulation of the acid–base balance of an organism [1]. Besides the volatile component CO_2_, nonvolatile acids are generated during metabolism, which cannot be eliminated by the lungs [2]. To maintain acid-base homeostasis, these nonvolatile acids must be excreted, and the filtered bicarbonate reabsorbed by the kidney. The renal regulation mechanisms thus include the regeneration of bicarbonate (HCO_3_ˉ) and the elimination of hydrogen ions (H^+^) from nonvolatile acids in free and fixed forms, especially titratable acids and ammonia, into the urine. 

Risk factors for chronic kidney disease (CKD) include race, gender, age, and family history. Moreover, smoking, obesity, hypertension, diabetes mellitus, systemic inflammatory state/inflammation, and urolithiasis can also lead to CKD [3,4,5]. The development of CKD is associated with significant changes in the acid excretion through the kidneys. With decreasing glomerular filtration rate (GFR), the decline in the renal capacity to synthesize ammonia and excrete hydrogen ions results in an increase in the prevalence of metabolic acidosis in CKD [6,7]. 

## 2. Metabolic Acidosis

Metabolic acidosis is an acid–base disorder, which is characterized by an imbalance in acid production relative to excretion, resulting primarily in an initial decrease in serum bicarbonate concentration [8]. Clinically, metabolic acidosis is defined as serum bicarbonate concentration <22 mmol/L [1,9]. However, acid retention can occur even when the serum bicarbonate level is apparently normal [10]. Chronic metabolic acidosis is common in advanced stages of CKD. A serum bicarbonate <22 mmol/L has been found in approximately 19% of patients with a GFR of 15–29 mL/min/1.73 m^2^ [11]. The clinical consequences of chronic metabolic acidosis include increased muscle protein catabolism and reduction of protein synthesis [11,12,13], disturbed bone metabolism [13,14], and increased mortality [15]. Metabolic acidosis is also known to cause insulin resistance, impaired thyroid hormone and growth hormone secretion [13]. The treatment of acidosis may reduce protein catabolism and loss of muscle mass and improve bone metabolism [13,16,17,18].

Moreover, studies suggest that chronic metabolic acidosis may be associated with further deterioration and progression of CKD and that correction of acidosis may improve kidney function and slow the progression of CKD [19]. The serum bicarbonate concentration is regarded as an independent predictor of CKD progression. A retrospective cohort study in 5422 adults showed that low serum bicarbonate level was associated with progression of kidney disease independent of baseline estimated GFR (eGFR) and other clinical, demographic and socioeconomic factors [20]. Patients in the lowest quartile of serum bicarbonate levels (≤22 mmol/L) at baseline had a 54% increased hazard of progression of kidney disease compared with those with serum bicarbonate concentrations of 25 to 26 mmol/L (reference group) after adjustment for multiple risk factors for progression. A randomized trial in 134 adult patients with CKD and serum bicarbonate levels of 16 to 20 mmol/L showed that supplementation with oral sodium bicarbonate slowed the rate of progression of renal failure to ESRD and improved nutritional status among these patients [21]. However, in a large CKD cohort, persistent serum bicarbonate >26 mmol/L was associated with increased risk of heart failure events and mortality [22].

As a progression factor of CKD, chronic metabolic acidosis requires correction. In patients with CKD and serum bicarbonate concentrations <22 mmol/L, clinical practice guidelines recommend alkali therapy to maintain the value within the normal range [9,23]. The prevention and treatment of chronic metabolic acidosis should primarily be achieved through diet modifications. Changes in dietary intake are reasonable even in patients with moderate renal impairment, because the high protein content of the usual diets may contribute to an increased proportion of acid equivalents.

## 3. Dietary Acid Load

Diet is known to be the most important individual factor that affects acid–base status [24,25,26,27]. The typical high-animal protein Western diets yield about 1 mmol/kg body weight/day of net endogenous H^+^ production [28,29], mostly a result of metabolizing the sulfur-containing amino acids methionine and cysteine [27]. On the other hand, base is generated from the metabolism of organic anions such as citrate and malate, which are contained in fruits and vegetables. The total net acid excretion (NAE) is analytically quantified from the 24-h urinary excretion of ammonium and titratable acid minus bicarbonate [24]. A controlled study in healthy adults consuming various diets revealed a strong relationship between the total renal NAE and pH value in 24-h urine. The analytically determined NAE was found to clearly reflect renal acid load in healthy subjects [27].

Moreover, the total urinary NAE can be reasonably estimated from dietary intake, intestinal absorption and the metabolism of the quantitatively in urine most important inorganic anions and cations. On the basis of these factors, the potential renal acid load (PRAL) can be calculated directly from dietary intakes [25,27]. The PRAL value indicates the calculated acid-forming potential (positive value) or base-forming potential (negative value) of single foods or diets on acid-base status. In Table 1 the average PRAL values of certain food groups are presented [25]. While protein-rich foods, such as meat, meat products, fish, and cheese are the food groups with the highest acid loads, fruits, vegetables, salads and fruit juices have a high alkalizing potential [27].

## 4. Net Acid Excretion and Urinary pH

Previous studies showed that the analytically determined NAE corresponded reasonably well to urinary pH value and the estimated NAE. On the basis of these findings, the PRAL value can be used for the compilation of definite diets [27,30]. The findings provide evidence that adjustments or specific manipulations of urinary pH and NAE to changes in dietary intake are possible [27]. A study in healthy adults revealed the lowest NAE after consumption of a lacto-vegetarian diet, a medium NAE under a moderate protein-containing diet and the highest NAE under a protein-rich diet [27]. An interventional study in healthy subjects confirmed that dietary pattern and urinary pH are closely linked together [30]. The protein-rich Western diet yielded lower urinary pH due to net production of H^+^ when metabolized. The shift to a nutritionally balanced mixed diet and an ovo-lacto-vegetarian diet, respectively, increased urinary pH due to the greater proportion of base-producing food components like fruits and vegetables [30]. 

**Figure 1:**
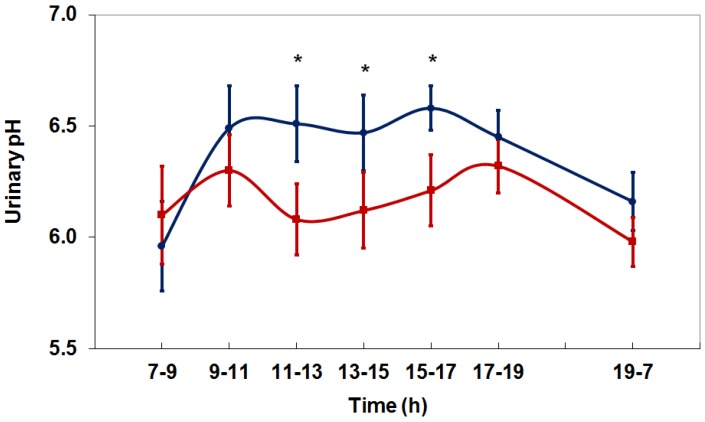
Diurnal variation in urinary pH during a 24-h period in healthy individuals under controlled, standardized conditions before (control, blue line) and after receiving 1500 mg/day l-methionine (red line) (M ± SEM) (* *p* < 0.05) [31].

A recent study in healthy adults showed that the administration of 1500 mg/day l-methionine clearly decreases diurnal urinary pH (Figure 1) [31]. Urinary sulfate excretion, a direct marker for the metabolism of l-methionine, and ammonium excretion, which reflects the increase in net acid production, increased significantly after l-methionine administration. The application of 1500 mg l-methionine per day corresponds to the methionine intake from about 200 g of chicken, cheddar cheese, or salmon, respectively, or from about 250 g of beef fillet or ham, respectively, according to food-specific data on methionine contents in databases [32].

## 5. Beverages

### 5.1. Fruit Juices

The effect of fruit juices on acid-base status is mainly determined by the presence of citrate. Citrus juices are rich sources of citrate [33]. Dietary citrate is absorbed in the intestine and nearly completely metabolized to bicarbonate, which in turn increases urinary pH [34]. Accordingly, the intake of 0.5 or 1.0 L/day orange juice under controlled conditions led to a significant increase in 24-h urinary pH in healthy adults, reflecting the alkalizing effect of orange juice [35]. However, due to the high energy, sugar and potassium content, the consumption of fruit juices should be limited.

### 5.2. Bicarbonate-Rich Beverages

**Figure 2:**
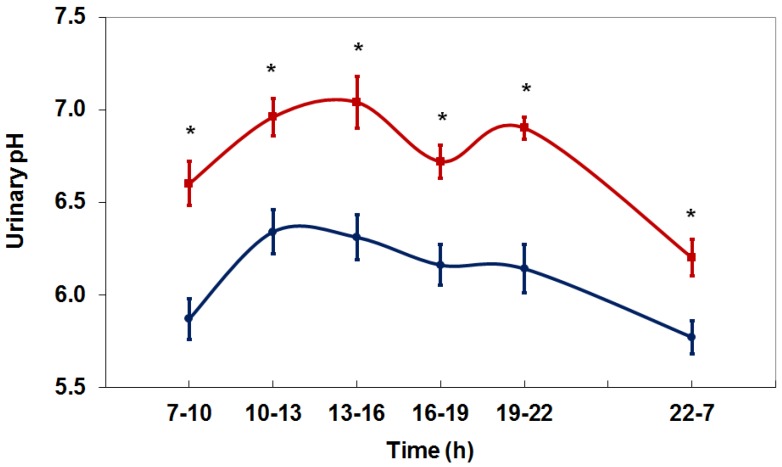
Diurnal variation in urinary pH during a 24-h period in healthy individuals under controlled, standardized conditions before (control, blue line) and after receiving bicarbonate-rich mineral water (red line) (M ± SEM) (* *p* < 0.05) [36].

The ingestion of bicarbonate increases the buffering capacity of the organism and has a strong alkalizing effect. Bicarbonate is a natural component of mineral water. A study in healthy subjects under controlled, standardized conditions revealed a significant increase in 24-h urinary pH from 6.10 to 6.59 after the intake of 1.4 L/day of a mineral water containing 3388 mg/L bicarbonate [36]. A persistent increase in the diurnal urinary pH was achieved through the evenly distribution of the fluid intake over the day (Figure 2).

Bicarbonate in mineral water can increase urinary pH as effectively as a medical therapy with alkali citrate. A randomized, cross-over study in healthy subjects compared the effect of a bicarbonate-rich mineral water and a commercial alkali citrate preparation on urinary pH [37]. The alkalizing effect of bicarbonate-rich mineral water was found to be similar to that of potassium citrate, which was administered in equimolar concentration with respect to the alkali load. The effect of water corresponds to that of alkali citrate or sodium bicarbonate in galenic form [38].

## 6. Dietary Acid Load and Risk of Chronic Kidney Disease

Prior studies have demonstrated that dietary acid-loading increased the risk and progression of CKD. In a cohort study of 12,293 adults, greater dietary acid load, quantified by estimated NAE, was associated with albuminuria and low eGFR, markers of CKD [39]. In 4564 participants in the Tehran Lipid and Glucose Study, participants in the highest quartile of PRAL had a 42% higher risk of CKD compared to the lowest quartile, independently of age, sex, body mass index and other confounding factors [40]. Analyses of Atherosclerosis Risk in Communities Study participants without CKD at baseline revealed that higher dietary acid load, estimated using the equation for PRAL, was associated with higher risk of incident CKD, even after adjusting for demographics and established risk factors, including diabetes, hypertension and overweight [41].

## 7. Diet versus Supplementation

An alkali therapy can not only be met by an alkali-rich diet but also by oral supplementation with alkali salts, e.g., sodium bicarbonate or alkali citrate [37,42]. A prospective, randomized, placebo-controlled, and blinded interventional study in 120 patients with hypertensive nephropathy with reduced but relatively preserved eGFR (mean 75 mL/min) compared the effect of daily oral sodium bicarbonate, sodium chloride or placebo, respectively [43]. After 5 years, the rate of eGFR decline was slower and eGFR was higher in patients given sodium bicarbonate than in those given placebo or equimolar sodium chloride. 

Since serum bicarbonate concentrations <22 mmol/L are associated with increased risk of CKD progression and mortality [15,20], treatment with oral bicarbonate supplementation should be applied to maintain serum bicarbonate within the normal range. Overtreatment to serum bicarbonate concentrations outside the normal range should be avoided, because high serum bicarbonate concentrations >26 mmol/L seem to be associated with increased risk of death irrespective of the level of kidney function [8,22]. 

In patients with CKD stage 4 due to hypertensive nephropathy, one year of dietary acid reduction with fruits and vegetables or sodium bicarbonate improved metabolic acidosis and reduced kidney injury without producing hyperkalemia [44]. A randomized study in 108 patients with stage 3 CKD (eGFR 30–59 mL/min/1.73 m^2^) and plasma total CO_2_ 22–24 mmol/L examined the effect of 36 months of dietary acid reduction by 50% using oral sodium bicarbonate or base-producing fruits and vegetables compared to usual care on GFR [42]. Fruits that were provided predominantly were apples, apricots, oranges, peaches, pears, raisins and strawberries. Vegetables that were provided predominantly were carrots, cauliflower, eggplant, lettuce, potatoes, spinach, tomatoes and zucchini. The study revealed that three years of dietary acid reduction with sodium bicarbonate or base-producing fruits and vegetables reduced urine excretion of angiotensinogen, an index of kidney angiotensin II levels, and preserved eGFR. The study supported that dietary acid reduction is kidney protective in CKD patients with metabolic acidosis. Since many fruits and vegetables are relatively rich in potassium, patients with impaired renal function should receive comprehensive dietary counseling, which should also be followed closely by the patient care providers for CKD patients to avoid electrolyte alterations such as hyperkalemia.

## 8. Conclusions

The studies suggest that kidney protective effects of an alkali therapy can be achieved through oral supplementation of sodium bicarbonate as well as through a base-producing diet. Primarily, the prevention and treatment of chronic metabolic acidosis should be realized by dietary modifications, because the high protein content of the usual diet contributes an increased proportion of acid equivalents. The primary goal of dietary treatment should be to increase the proportion of fruits and vegetables and to reduce the daily protein intake to 0.8–1.0 g per kg body weight. Since an inadequate diet could lead to micronutrient and macronutrient deficiencies [45], protein malnutrition must be avoided. 

## Figures and Tables

**Table 1:** Average potential renal acid loads (PRAL) of certain food groups (related to 100 g edible portion) [27].

Food Group	PRAL (mEq/100 g)
Fruits and fruit juices	−3.1
Vegetables	−2.8
Fats and oils	0
Milk and whey based products	+1.0
Bread	+3.5
Noodles, spaghetti	+6.7
Fish	+7.9
Cheese (protein <15 g/100 g)	+8.0
Meat and meat products	+9.5
